# Assessment of Chemical and Biological Fungicides for the Control of *Diplodia mutila* Causing Wood Necrosis in Hazelnut

**DOI:** 10.3390/plants13192753

**Published:** 2024-09-30

**Authors:** Verónica Retamal, Juan San Martín, Braulio Ruíz, Richard M. Bastías, Eugenio Sanfuentes, María José Lisperguer, Tommaso De Gregorio, Matteo Maspero, Ernesto Moya-Elizondo

**Affiliations:** 1 Departamento de Producción Vegetal, Facultad de Agronomía, Universidad de Concepción, Chillán 3812120, Chile; veronicaretamal@udec.cl (V.R.); juansanmartinm@udec.cl (J.S.M.); braruiz@udec.cl (B.R.); ribastias@udec.cl (R.M.B.); 2 Laboratorio de Patología Forestal, Facultad de Ciencias Forestales y Centro de Biotecnología, Universidad de Concepción, Concepción 4030000, Chile; esanfuen@udec.cl; 3 Departamento Técnico, Frutícola Agrichile S.A., Curicó 3340000, Chile; mjlisperguer@agrichile.cl; 4 Agri Competence Centre, Ferrero Hazelnut Company (HCo), Senningerberg, L-2633 Luxembourg, Luxembourg; tommaso.degregorio@ferrero.com (T.D.G.); matteo.maspero@ferrero.com (M.M.)

**Keywords:** *Corylus avellana* L., fungal trunk disease, fungicides, biocontrol agents, *Pseudomonas protegens* ChC7, *Bacillus subtilis* QST 713

## Abstract

Fungal trunk disease (FTD) poses a significant threat to hazelnut (*Corylus avellana* L.) production worldwide. In Chile, the fungus *Diplodia mutila*, from the Botryosphaeriaceae family, has been frequently identified causing this disease in the Maule and Ñuble Regions. However, control measures for *D. mutila* remain limited. This research aimed to evaluate the effectiveness of chemical and biological fungicides against *D. mutila* under in vitro, controlled pot experiment, and field conditions. An in vitro screening of 30 fungicides was conducted. The effectiveness was assessed by measuring the length of vascular lesions in hazelnut branches inoculated with *D. mutila* mycelium disks under controlled and field conditions. Field trials were conducted in a hazelnut orchard in Ñiquén, Ñuble Region, Chile. The results showed that three biological and five chemical fungicides were selected in vitro with >31% inhibition after 14 days. In pot experiments, all fungicides reduced necrotic lesions on branches by 32% to 61%. In field experiments, the most effective systemic fungicides were fluopyram/tebuconazole, fluxapyroxad/pyraclostrobin, and tebuconazole, while the effectiveness of antagonists *Pseudomonas protegens* ChC7 and *Bacillus subtilis* QST713 varied with seasonal temperatures. Effective conventional and biological fungicides against *D. mutila* could be integrated into disease management programs to protect hazelnut wounds from infections.

## 1. Introduction

The cultivated area of European hazelnut (*Corylus avellana* L.) has increased significantly in Chile over the last ten years, reaching 36,375 hectares [1] which represents 9.7% of the total land surface used for fruit production, ranking hazelnut as the fifth most important fruit crop in the country. Several diseases caused by bacteria and fungi have become more recurrent in this nut fruit crop. On a national scale, there are reports of bacterial diseases such as crown gall caused by *Agrobacterium tumefaciens*; bacterial canker caused by *Pseudomonas avellanae*, *P. syringae* pv. *syringae*, and *P. syringae* pv. *coryli*; and bacterial blight caused by *Xanthomonas arboricola* pv. *corylina*; while reports of fungal diseases include root rot caused by *Armillaria mellea*; and wood canker by *Dothiorella vidmadera* (=*Diplodia coryli*) [2]. In recent years, a higher incidence of diseases affecting the woody tissues of the plant has been reported in hazelnut and other important fruit species in Chile, such as walnut, kiwi, and grapes [3,4,5]. Furthermore, environmental conditions and climate change are recognized predisposing factors for these types of diseases [6].

Different pathogens causing wood damage in hazelnut have been reported in Italy [7,8], the United States [9], and Chile [10,11,12,13,14], with symptoms such as cankers, vascular discoloration, and regressive death of the trunk, branches, twig, or the entire plant. This syndrome has been called fungal trunk disease (FTD) in hazelnut and other species [8]. These wood pathogens include members of the Botryosphaeriaceae family, with around 300 species, some of which are recognized for affecting numerous woody hosts in temperate and tropical regions [15]. Botryosphaeria pathogens enter through natural openings and wounds resulting from management practices in the orchard, where lesions are evident in the lateral sections of affected branches, typically expanding along the xylem [16,17]. Within this family, species of the genera *Diplodia* and *Dothiorella* have been mainly described in hazelnut [9,11,14,18]. In Chile, *Diplodia coryli* Fuckel, reclassified as *Dothiorella vidamera* W.M. Pitt, J.R. Úrbez-Torres and Trouillas sp. nov. [19], has been reported causing greyish discoloration in wood and regressive death in five-year-old plants of cv. Barcelona [11], while vascular necrosis caused by *Diplodia mutila* (Fr.) Mont (teleomorph *Botryosphaeria stevensii* Shoemaker, [1964]) was first reported in hazelnut orchards in Oregon, USA [9], and recently in the Maule and Ñuble Regions, Chile [14]. This fungal pathogen has also been described causing vascular discoloration, cankers on the branches and trunk, and death of the small branches in apple trees [20], grapevines [21], walnut trees [22], and *Araucaria araucana* [23] in Chile. Despite the impact of plant pathogens such as *D. mutila*, research has mainly focused on agricultural practices such as the selection of healthy plants, pruning of infected tissues, and tool disinfections, while there are no studies addressing control strategies for these pathogens in hazelnut [2,24].

Fungicides such as fluazinam, carbendazim, methyl thiophanate, fludioxonil, pyraclostrobin, myclobutanil, penconazole, and tebuconazole have shown high efficacy on isolates of *D. mutila* from grapevines under in vitro conditions [25]. However, the main buyer of European hazelnuts produced in Chile maintains strict restrictions on the use of chemical fungicides [26], and thus biological control agents (BCAs) can play an important role in the control of *D. mutila*. However, few studies have evaluated the direct effect of BCAs on *D. mutila*. There is evidence that fungi such *Acremonium mucronatum* significantly reduced mycelial growth of *D. mutila* in vitro and on branches of Quercus trees [27], while other studies on the biocontrol activity of *Trichoderma harzianum* Th-1 INTA showed parasitism on *D. mutila* and a marked inhibitory effect on conidia germination and pycnidium formation under in vitro conditions [28]. On the other hand, isolates of *Pseudomonas putida* JRSK-39, *P. koreensis* JRSK-45, and *Bacillus halotolerans* JFM-64, which are rhizobacteria isolated from grapevine, have shown significant reductions in the mycelial growth of *D. mutila* [29], while research on *P. aurofaciens* has demonstrated a reduction in necrotic lesions caused by *D. mutila* on stems of *Fraxinus excelsior* plants [30]. In Chile, it has been described that the aging of hazelnut orchards results in a higher detection of fungal phytopathogen species causing cankers, vascular necrosis, and cambium death [13,14].

Therefore, the objective of this study was to assess the effectiveness of chemical and biological fungicides against *D. mutila* under in vitro, controlled, and field conditions.

## 2. Results

### 2.1. Selection of Active Ingredients of Chemical and Biological Fungicides

After 48 h of incubation, nine products (43%) inhibited the growth of *D. mutila* between 76% to 99% (+++) (Table 1). However, fifteen of the products evaluated were discarded after 120 h of incubation, while only thiophanate-methyl, fluxapyroxad/pyraclostrobin, fluopyram/tebuconazole, fluazinam, tebuconazole, and prochloraz showed inhibition of the fungal colony between 31% and 75%, which had a qualitative scale value of (++). After 14 days, fungal growth was reactivated in most products, while fungal inhibition was maintained in PDA plates treated with prochloraz, fluazinam, tebuconazole, fluxapyroxad/pyraclostrobin, and fluopyram/tebuconazole.

**Figure 1:**
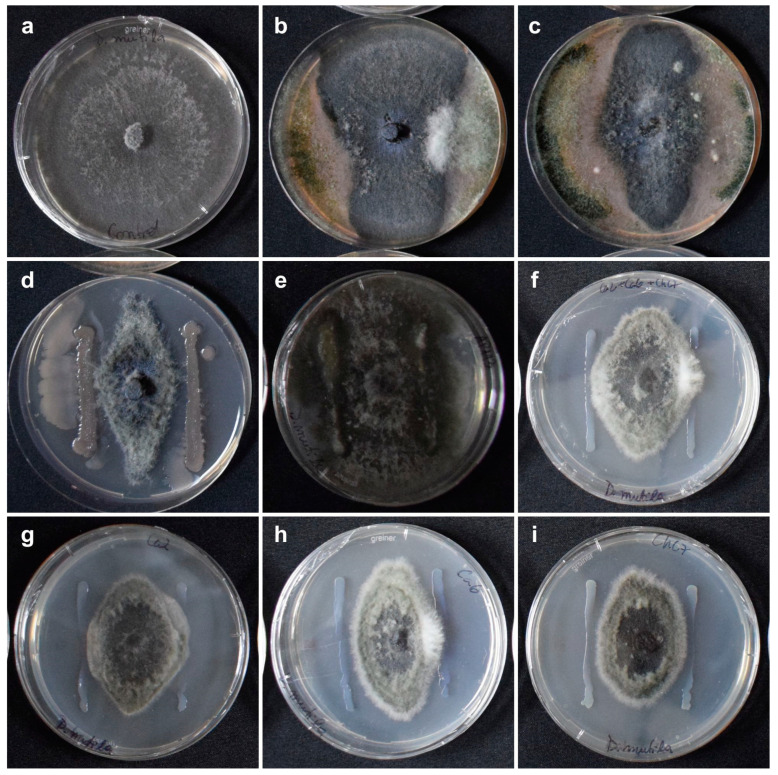
Pre-selection assay of antagonist microorganisms against *Diplodia mutila* after 7 days of incubation. (**a**) Control: *D. mutila*; (**b**) *Trichoderma* spp. and *Bacillus* spp. (Puelche-VTO, Bio Insumos Nativa SpA); (**c**) *Bionectria ochroleuca* Mitique, *Trichoderma gamsii* Volqui, *Hypocrea virens* Ñire (Mamull, Bio Insumos Nativa SpA); (**d**) *Bacillus subtilis* QST 713 (Serenade ASO, Bayer de México, S.A, Tiaxcala, Mexico); (**e**) *Pantoea* sp. AP113; (**f**) *Pseudomonas protegens* Ca2 + Ca6 + ChC7; (**g**) *P. protegens* Ca2; (**h**) *P. protegens* Ca6; and (**i**) *P. protegens* ChC7.

Among the antagonist bacteria, significant differences were observed (Kruskal–Wallis, *p* = 0.02). *Bacillus subtilis* QST713 showed the greatest inhibition of *D. mutila* colony growth (52%), followed by *Pseudomonas protegens* ChC7, while *Pantoea* sp. AP113 and *P. protegens* Ca6 had lower efficacy, with 6.3% and 38%, respectively (Table 2; Figure 1).

In the evaluation of the degree of antagonism, the biological fungicide based on a consortium of *Bionectria ochroleuca* Mitique, *Trichoderma gamsii* Volqui, *Hypocrea virens* Ñire (Mamull^®^ WG. Bio Insumos Nativa SpA, Maule, Chile) outperformed *D. mutila* and covered at least two-thirds of the surface of the medium (Class 2), classifying it as an antagonist agent [31]. On the contrary, the biological product based on *Trichoderma* spp. and *Bacillus* spp. (Puelche—VTO, Bio Insumos Nativa SpA, Maule, Chile) allowed the colonization of less than a third of the surface of the medium (Class 4), which is not considered antagonistic (Figure 1).

### 2.2. Assessment of Effectiveness of Chemical and Biological Fungicides against Diplodia mutila in Hazelnut Plants under Pot-Controlled Conditions

The average length of necrotic lesions (mm) for each treatment against *D. mutila* is shown in Table 3. No interaction was observed between treatment and cultivar factors (*p* = 0.91), and there were no differences among cultivars (*p* = 0.87), but significant differences were observed among the treatments applied to the holes inoculated with the fungus (*p* < 0.01; C.V. = 9.5). Compared to the untreated control, all chemical and biological fungicides significantly reduced the necrosis associated with the development of the fungus and the death of internal branch tissues, with a reduction ranging between 32% and 61%. The most effective treatments were fluxapyroxad/pyraclostrobin, fluazinam, the consortium of *B. ochroleuca* Mitique, *T. gamsii* Volqui, and *H. virens* Ñire, and fluopyram/tebuconazole, with no significant differences among them. Additionally, the antagonists *B. subtilis* QST 713 and *P. protegens* ChC7 showed no statistical differences compared to fluopyram/tebuconazole and prochloraz (Table 3; Figure S4).

### 2.3. Assessment of Effectiveness of Chemical and Biological Fungicides against Diplodia mutila under Field Conditions

Pearson’s analysis determined that there was no correlation (*p* > 0.05) between branch diameter (mm) and length of vascular necrosis (mm) caused by the fungus in the two seasons under study (r = 0.20 [*p* = 0.32] for the 2020–2021 season and r = 0.05 [*p* = 0.80] for the 2021–2022 season).

During the 2020–2021 season (Table 4), no interaction was detected between the factors of chemical treatments and inoculation time (*p* = 0.27; C.V. = 6.34). Furthermore, there were no significant differences in the length of vascular necrosis caused by *D. mutila* in branches treated with chemical fungicides (*p* = 0.82). However, significant differences were detected among the average lengths of vascular necrosis at each inoculation time (*p* > 0.01), indicating that branches inoculated with *D. mutila* on the same day as application presented greater necrotic lesions (Figure S5).

**Figure 2:**
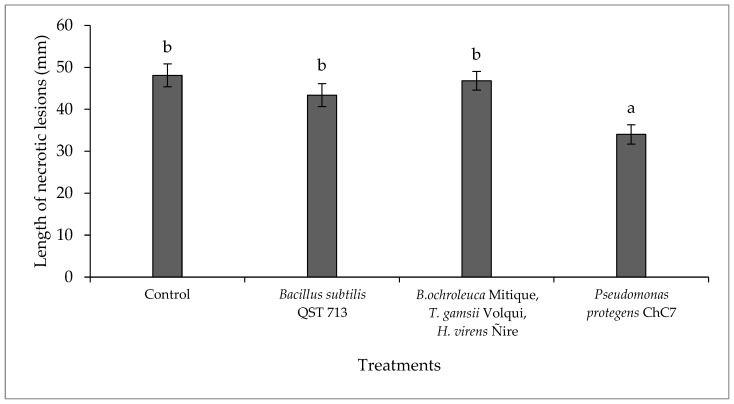
Mean length values of necrotic lesions caused by the fungus *Diplodia mutila* on branches of hazelnut cv. Tonda di Giffoni, inoculated the same day and 24 h after the application of biological control agents, under field conditions during the 2020–2021 season. Bars with different letters indicate statistical differences between treatments according to Fisher’s LSD test (α = 0.05).

There was no interaction between the factors of biological treatments and inoculation time (*p* = 0.41; C.V. = 4.56). Differences were observed among lesions in branches treated with biological control agents (*p* > 0.01; Figure 2 and Figure S8), where in the presence of the antagonist *P. protegens* ChC7, a 29.2% reduction in necrosis was observed compared to the control (Figure S8). There were no differences between inoculation times (*p* = 0.79).

In the 2021–2022 season (Table 5; Figures S6 and S7), there was an interaction between the factors of chemical treatments and inoculation time (*p* < 0.01; C.V. = 2.75). Additionally, each factor showed significant differences (*p* < 0.01). Except for fluazinam, chemical fungicides significantly reduced the length of the lesion caused by *D. mutila* when inoculated on the same day as treatment application, ranging from 24% to 37% compared to the control. However, in the inoculation conducted 24 h after treatment application, only the systemic fungicides tebuconazole, fluxapyroxad/pyraclostrobin, and fluopyram/tebuconazole showed significant differences, ranging from 29% to 38% compared to the control (Figures S6 and S7). Additionally, significant differences were observed among inoculation times (*p* < 0.01), with the length of vascular necrosis being lower in all treatments of the first inoculation compared to the second inoculation.

**Figure 3:**
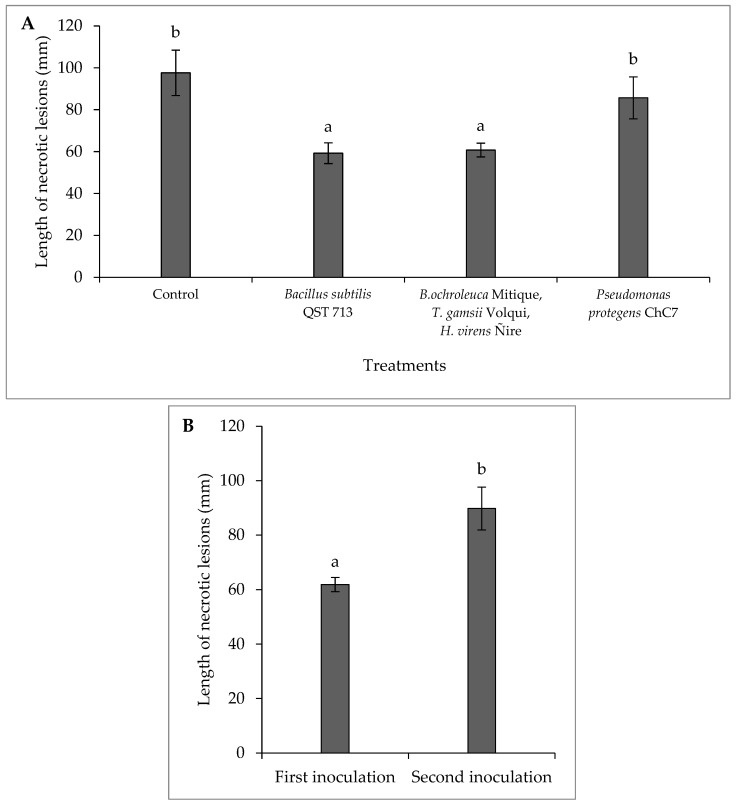
(**A**) Mean length values of necrotic lesions caused by the fungus *Diplodia mutila* on branches of hazelnut cv. Tonda di Giffoni; (**B**) inoculations on the same day (first inoculation) and 24 h later (second inoculation) after the application of biological control agents, under field conditions in the 2021–2022 season. Bars with different letters indicate statistical differences between treatments (**A**) and inoculation times (**B**) according to Fisher’s LSD test (α = 0.05).

There was no interaction between the factors of biological treatments and inoculation time (*p* = 0.19; C.V. = 3.89). Differences were observed among lesions in branches treated with biological control agents (*p* < 0.01; Figure 3 and Figure S9), where in the presence of the antagonist *B. subtilis* QST 713 and the consortium of fungi (*B. ochroleuca* Mitique, *T. gamsii* Volqui, and *H. virens* Ñire), reductions of 38% and 40%, respectively, were observed (Figure 3 and Figure S9). Additionally, there were differences between inoculation times (*p* < 0.01), with a 33% increase in necrotic lesions in the second inoculation.

Based on the number of catkins and glomerules evaluated, the inoculated and treated branches did not affect the fruiting potential of the species when evaluated as a variation in the density of male and female flowers (Table S2).

## 3. Discussion

*Diplodia mutila* is a phytopathogenic fungus primarily associated with wood diseases in agricultural crops and forestry species of interest [9,14,22,23,32,33,34,35]. This study represents the first investigation into the effectiveness of chemical and biological fungicides in reducing the damage caused by this fungus in European hazelnut. Considering the recent report of *D. mutila* affecting European halzelnut in Chile [14], there are no current methods or registered fungicides to control this pathogen in Chile. Of the nineteen chemical fungicides evaluated in vitro, using a quick screening test by using recommended commercial doses in other fruit crops, only six qualitatively reduced the development of *D. mutila* when applied at commercial doses on the culture medium. In particular, methyl thiophanate was highly toxic on *D. mutila*, which agrees with the findings of Mondello et al. (2018) [25] and Sosa et al. (2022) [28]. However, due to the European Commission’s decision not to renew approval for this active substance based on the latest report [36], which highlighted insufficient information regarding its safe use in the environment and with regard to mammals, methyl thiophanate was excluded from the plant trials in this research.

When studying the effective concentration of the other selected fungicides, fluazinam and the combinations of fluxapyroxad/pyraclostrobin and fluopyram/tebuconazole showed greater inhibitory effects on mycelial growth. The active ingredients belonging to the demethylation inhibitor (DMI) groups, tebuconazole and prochloraz, showed mycelial inhibition results in isolate F096 as have been reported on other Chilean isolates of *D. mutila* obtained from grapevines [37].

The combinations of active ingredients with different action mechanisms, fluopyram/tebuconazole and fluxapyroxad/pyraclostrobin, showed greater toxicity against *D. mutila*. The efficacy of tebuconazole and pyraclostrobin against *D. mutila* species has been widely reported in the literature [25,37,38,39]. To the best of our knowledge, there is no information on the effectiveness of fluopyram and fluxapyroxad against *D. mutila*. Even though sole active ingredients were not evaluated, it was determined that the combination of fluopyram/tebuconazole was more toxic than using tebuconazole alone, even at 50% less concentration, which suggests a significant fungicidal effect of fluopyram. In the case of pyraclostrobin, a product based solely on this active ingredient (Comet^®^, BASF, Guaratinguetá, Brazil) was discarded in the qualitative selection assay because of its low inhibitory effect under in vitro conditions, suggesting a greater inhibitory effect on *D. mutila* when combined with fluxapyroxad.

An assessment of effective concentration showed that the six selected active ingredients had high efficacy at the commercial dose assessed, providing a broad range for evaluating control doses under field conditions. In this sense, EC50 determination is essential to assess and/or monitor changes in fungicide sensitivity in populations of less sensitive pathogens and to make informed choices on the use of active ingredients [40]. Additionally, determination of EC50 will allow determining lower doses for chemical fungicides to reduce potential effects on non-target fungal populations, as described by de la Cruz et al. (2022) [41].

In the present research, the commercial dose of synthetic fungicides recommended by manufacturers was used to quickly discard a large number of products since those doses have shown efficacy in controlling other fungi, and the absence of phytotoxicity in other fruit tree species. The application of the effective concentration against *D. mutila* is important, particularly considering that this pathogen has been recently detected in hazelnut [9,14], and that there are no reports on fungicides that can control this fungus in European hazelnut.

Inhibition rates demonstrated the antagonistic effect of Chilean strains of *Pseudomonas protegens* on *D. mutila*. Among these strains, *P. protegens* ChC7 was the most effective and has been available in the local market since 2022 (TANIRI^®^ WP, Bio Insumos Nativa SpA, Chile) due to its ability to induce plant resistance [42,43]. Previous research has shown that these *P. protegens* strains, isolated from the wheat rhizosphere, exhibit antagonistic activity against other phytopathogenic species affecting important crops in Chile [42,44,45]. This activity is associated with the synthesis of secondary metabolites, including antibiotics such as 2,4-diacetylphloroglucinol (2,4-DAPG), pyrrolnitrin, and pyoluteorin, which are produced by the genes *phl*, *prn*, and *plt*, respectively [42,44,45,46]. Additionally, this bacterial species can induce plant resistance [43,47]. In a study on the control of necrotic wood fungi, soil rhizobacteria isolates from grapevines showed effective results in inhibiting the mycelial growth of *D. mutila* compared to various *Pseudomonas* species, using a methodology similar to that used in this study [29]. Furthermore, Huang et al. (2022) [48] determined the efficacy of *P. protegens* in reducing mycelial growth and postharvest rot caused by a Botryosphaeriaceae fungus like *Botryosphaeria dothidea*, the causal agent of apple ring rot. In our study, *Bacillus subtilis* QST 713 exhibited the highest inhibitory effect on the mycelial growth of *D. mutila* among the tested antagonistic bacteria in vitro. This *Bacillus* strain is widely used as a biological control agent (BCA) due to its broad-spectrum inhibitory activity against several phytopathogens through the production of antibiotics and antifungal enzymes [49], which provides evidence of its inhibitory effect in vitro on the mycelial growth of *D. mutila*. Moreover, it can induce systemic acquired resistance (SAR) in plants [50].

The consortium of *B. ochroleuca* Mitique, *T. gamsii* Volqui, and *H. virens* Ñire (anamorph: *Trichoderma virens*) showed the highest efficacy in the in vitro experiments, although there are few published reports on its use against wood-infecting pathogens. Silva-Valderrama et al. (2021) [51] demonstrated that this consortium restricted over 50% of the mycelium area of *D. seriata*, *Neofusicoccum parvum*, and *Phaeomoniella chlamydospora* obtained from grapevines on PDA plates. Additionally, Mondello et al. (2018) [25] showed that an isolate of *T. gamsii* was effective in inhibiting the teleomorph of *D. mutila* in in vitro studies, which is associated with possible antibiosis and mycoparasitism [52]. These findings agree with the in vitro results obtained in the present study, highlighting the potential of BCAs like *Trichoderma* for the control of pathogens causing wood necrosis in European hazelnut.

The controlled pot experiment validated the efficacy of chemical fungicides against *D. mutila* observed in the in vitro assays based on a quick screening test, as all five products effectively reduced necrotic lesions on the branches of inoculated nursery plants. The manufacturer’s recommended dose for chemical fungicides considers concentrations that are non-phytotoxic to a variety of crops, including vegetables, industrial crops, and fruit and forest trees (Table S3). Therefore, the dose used for fruit trees was applied. The concentration of the active ingredient (mg L^−1^ or ppm) in the commercial dose varied for each fungicide, reaching concentrations within the treated and subsequently inoculated hole wound with the pathogen ranging from 0.05 mg (tebuconazole) to 0.14 mg (prochloraz) (Table S4). However, the observed high efficacy of the commercial doses of the five chemical fungicides and the three biofungicides against *D. mutila* needs validation for their efficacy against other wood-necrotizing fungal species such as *D. coryli* [11], *Diaporthe australafricana* [53], and *Neofusicoccum parvum* [12], which have been reported affecting European hazelnut in Chile.

In general, there is limited information on the epidemiology and etiology of *D. mutila* infection in European hazelnut. According to a comprehensive study and review of vine disease management conducted by Gramaje et al. (2018) [54], Botryosphaeria dieback caused by botryosphaeria fungi is transmitted through airborne spore dispersal, with infection primarily occurring at cuts from annual pruning or any open wounds, followed by colonization and necrosis of vascular tissues. A monitoring study of *D. mutila* conidia conducted over two years in grapevines in France determined that conidia release was associated with rain episodes, and high-risk infection periods occurred from early to mid-fall, mid-spring, and late summer, with low conidia detection during winter [55]. Mycelial growth of *D. mutila* in vitro occurs within the range of 5 °C to 35 °C, with optimal growth between 20 °C and 30 °C and an optimum temperature of 25 °C [56]. Considering temperature variability during the month of treatment application in the field (October 2020), the environmental conditions recorded 30 days after plant inoculation had an average temperature of 12.8 °C, with an average minimum of 5.2 °C (Range: 0.8–10.7 °C) and an average maximum of 20.4 °C (Range: 16.2–26.8 °C) (Figure S10A). During the 208 days of the field experiment in the 2020–2021 season, temperatures above 20 °C were observed for 41 days. In the second season, over the 160-day period of the experiment, temperatures above 20 °C were recorded for 60 days, with a 30-day period post-inoculation having an average temperature of 15.5 °C and an average minimum of 7.5 °C (Range: 2.1–11.7 °C) and an average maximum of 23 °C (Range: 15.3–29.3 °C) (Figure S10B). Temperature variations between the two seasons could explain the differences in the length of necrotic lesions observed on branches inoculated with *D. mutila*, suggesting that longer necrotic lesions in the vascular tissue of branches inoculated in the 2021–2022 season were related to a more extended period of favorable temperatures for mycelial development. Moreover, regardless of the time in which the tissue remained infected with this pathogen, temperature would be a determining factor in the level of damage experienced by hazelnut tissues. On the other hand, the conditions in the second season favored the development of the fungus and allowed for significant differences between the treatments based on chemical and biological fungicides. Precipitation and relative humidity conditions did not vary significantly between the seasons and would not have influenced the observed necrosis levels for each treatment (Figure S10). This information provides insights into environmental conditions favoring pathogen infection and, combined with data on *D. mutila* spread, can help decision-making regarding fungicide application to protect the plants.

The results from both the pot and field experiments, particularly in the second season, confirm the efficacy of traditional chemical fungicides in mitigating the damage inflicted by *D. mutila* on hazelnut branches. Furthermore, active ingredients with systemic properties led to a notable decrease in necrotic lesions compared to contact-based products. In the case of branches inoculated 24 h post-fungicide application, tebuconazole, fluxapyroxad/pyraclostrobin, and fluopyram/tebuconazole demonstrated prolonged activity in the plant wound, resulting in a more pronounced reduction in pathogen-induced damage.

In the pot experiments, antagonistic organisms proved to be highly effective in disease control (30% to 55% efficacy). However, there was variability in the performance of biological treatments between the seasons in the field trials. Furthermore, it was evident that the impact of biological treatments persisted in both direct and deferred inoculations, revealing that beneficial microorganisms could reproduce in the tissues of hazelnut plants, achieving effective control of *D. mutila*. Only *P. protegens* ChC7 demonstrated efficacy in the 2020–2021 season, while *B. subtilis* QST 713 and the consortium of fungi *B. ochroleuca* Mitique, *T. gamsii* Volqui, and *H. virens* Ñire showed positive results in the 2021–2022 season. *P. protegens* is a bacterium isolated from wheat roots, and thus it grows at lower temperatures [42,44], which could explain its higher efficacy during the colder temperatures of the first season. In contrast, *B. subtilis* QST 713 and *Trichoderma* spp. or *Hyprocrea* spp. require higher temperatures (25–55 °C; 25–30 °C; 25–30 °C, respectively) for optimal antagonistic activity [36,57,58], which could account for their greater efficacy in controlling *D. mutila* during the second season.

The results of the floral density analysis (Table S2) demonstrate that the application of treatments did not affect the productive potential of the plant. These findings suggest that no damage is generated in the wood that could affect the floral development of both catkins and clusters in the early stages of infection caused by *D. mutila*. This might occur because floral induction in hazelnut occurs between 4 and 5 months before flowering, specifically during the months of December and January [59]. Given that the fungus was inoculated in early spring, the period of greatest damage caused by this pathogen does not necessarily coincide with the period of greatest susceptibility to negative effects on floral development. Another explanation could be associated with the source–sink relationship, as it has been determined that branches or floral shoots of hazelnut do not exhibit autonomy in their carbohydrate source–sink capacity and are essential for floral development [60]. Therefore, even though the damage caused by the fungus resulted in injury at the phloem level of the branch, it does not imply an interruption in the direct flow of carbohydrates for the development of new flowers for the following season.

Considering that the main market for the consumption and export of Chilean hazelnuts is Europe, the use of active substances for pest and disease control faces higher restrictions aimed at minimizing environmental impact. Therefore, antagonists (*Pseudomonas protegens* or *Bacillus* spp.) and active ingredients with systemic action (fluopyram/tebuconazole, fluxapyroxad/pyraclostrobin, and tebuconazole) would be effective tools in reducing damage caused by *D. mutila*. However, it is essential to conduct accurate monitoring of environmental conditions during crop production seasons, particularly rainfall and temperatures above 20 °C, as they favor the spread and development of this fungus. Similarly, environmental factors would influence the selection of antagonistic microorganisms to be included in a phytosanitary management plan that promotes their reproduction and control activity. The use of registered fungicides and BCAs is crucial for establishing integrated disease control and maintaining quality and safety in hazelnut production.

## 4. Materials and Methods

### 4.1. Phytopathogenic Fungus

The phytopathogenic fungus *Diplodia mutila* isolate F096 was selected due to its high aggressiveness on hazelnut (*Corylus avellana* L.) [14]. This isolate was collected from wood exhibiting vascular necrosis in 2018 from hazelnut orchards in the Ñuble Region, Chile. The phytopathogen was stored in the microorganism collection of the Plant Pathology Laboratory at the Faculty of Agronomy, Universidad de Concepción, Chillán Campus.

### 4.2. In Vitro Pathogen Control Assays

Thirty treatments were used to assess the efficacy in controlling *D. mutila* under in vitro conditions. The evaluated treatments included nineteen chemical fungicides with different modes of action, three natural extracts and salts, and eight biological control agents (BCAs), consisting of three commercial biopesticides and five bacterial microorganisms from the collection of the Plant Pathology Laboratory at the Faculty of Agronomy (Table S1).

### 4.3. Selection of Treatments with Fungicidal Activity against Diplodia mutila

Solutions were prepared using the commercial doses or concentrations suggested by the manufacturer for each fungicide (Table S1). For the chemical fungicides, a 500 µL aliquot of each product solution was evenly spread on a Petri dish (80 mm diameter) containing 10 mL of potato dextrose agar (PDA) medium. After one hour, once the medium surface was dry, 5 mm diameter discs of actively growing *D. mutila* mycelium were placed to assess the ability to inhibit fungal mycelial growth. The assessment was conducted at 48 and 120 h after incubation using a rating scale with five levels of mycelium growth on the PDA medium: (−) = normal fungal growth, (+) = moderate fungal growth (1% to 30% less than normal fungal growth), (++) = mild fungal growth (31% to 75% less fungal growth), (+++) = limited fungal growth (76% to 99%), and (++++) = 100% inhibition of fungal growth (Figure S1). Three repetitions were performed for each treatment. The plates were kept in an incubation chamber and incubated in the dark at 25 °C for two weeks.

For bacterial and fungal antagonists, commercial doses of bioproducts and suspensions of bacteria grown in King B (KB) broth from the laboratory collection were applied to the PDA medium using a 10 µL bacteriological inoculation loop. Two straight parallel lines were extended 15 mm from the 5 mm diameter mycelium disc of the target fungus, placed at the centre of the plate. Three repetitions were performed for each treatment. The plates were maintained in an incubation chamber at 25 °C in darkness for one week. *Pseudomonas protegens* bacterial strains were grown by taking 150 µL of the strain sample and cultured in KB broth at 25 °C for 48 h in a shaking incubator at 150 rpm. The bacterial concentration was determined by counting colony forming units (CFUs) in serial dilutions.

For the biological treatments based on antagonistic bacteria, fungal growth was determined by measuring the fungal colony mycelium diameter (mm) with a steel ruler (Hand^®^, 200 mm), while the action of products with *Trichoderma* species was evaluated using the antagonism classification proposed by Bell et al. (1982) [31], with the following growth patterns: Class 1 = *Trichoderma* completely overgrows the pathogen and covers the entire medium surface; Class 2 = *Trichoderma* overgrows at least two-thirds of the medium surface; Class 3 = *Trichoderma* and the pathogen each colonize approximately one-half of the medium surface (more than one-third and less than two-thirds), and neither organism dominates the other; Class 4 = the pathogen colonizes at least two-thirds of the medium surface and appears to withstand encroachment by the *Trichoderma* invasion, occupying the entire medium surface; Class 5 = the pathogen completely dominates *Trichoderma*, overgrows it, and occupies the entire medium surface. *Trichoderma* was considered a good antagonist if the average score for a given comparison was less than or equal to 2, but not very antagonistic if the number was greater than or equal to 3.

### 4.4. Assessment of Effectiveness of Chemical and Biological Fungicides against D. mutila in Hazelnut Plants under Controlled Conditions

The pot experiment to assess the preventive control of *D. mutila* with the selected fungicide treatments was conducted using two-year-old hazelnut plants of cv. Barcelona and Tonda di Giffoni (Figure S3). Plants with a central trunk axis of approximately 150 cm in height were maintained in individual plastic pots of 0.03 m^3^ with a substrate of peat moss and perlite (3:1).

A 6.5 mm diameter hole was drilled in the trunk of each plant using a drill (Bauker^®^, model SD-GS1041, Suzhou, China) to a depth of 4 mm (Figure S3). Subsequently, 300 µL of the chemical fungicide or BCA treatment selected from the in vitro assays was applied to an individual hole using a micropipette. After 20 min, the treated hole was inoculated with a 5 mm diameter mycelium disc of *D. mutila*, and additionally, a PDA disc was inoculated as a control in a separate hole. The inoculated wounds were sealed with plastic film throughout the experiment to prevent dehydration. Plants were maintained under a 50% shaded Raschel mesh cover and irrigated three times weekly for five months. The treatments were distributed in a randomized complete block design with a factorial arrangement, and the experiment had seven replications.

At the end of the pot experiment, the central axes of each plant were removed, and each branch or stem segment was cut vertically through the inoculation point to measure the length of the necrotic lesion (mm) and assess the effect of each treatment on *D. mutila* compared to the water control. Additionally, fungi were isolated from the lesions by cutting pieces of the advancing necrosis zone and disinfecting the surface for 1 min in a 2.5% sodium hypochlorite solution, followed by two washes with sterile distilled water (SDW) for 1 min. Each branch piece was placed on plates with PDA medium containing streptomycin (200 mg L^−1^). The plates were incubated in a growth chamber at 25 °C for 10 days in darkness. Fungi were identified based on their morphological characteristics observed using an optical microscope (Motic^®^ BA310E, Motic, Xiamen, China).

### 4.5. Assessment of Effectiveness of Chemical and Biological Fungicides against D. mutila under Field Conditions

#### 4.5.1. Study Area

The experiment was conducted in an eight-year-old hazelnut orchard of cv. Tonda di Giffoni over two consecutive seasons (2020–2021 and 2021–2022). The orchards were located in “Campo San Gregorio,” Ñiquén, Ñuble Region, Chile (36°18′25″ S; 71°49′32″ W), owned by Frutícola AgriChile S.A. The orchards were established in a multiple-leader trained system with a planting framework of 5 m between rows and 3 m between plants on the row, under drip irrigation and fertirrigation. Soils belonged to the Tuiquilemu series (Inceptisols) (orchard in the 2020–2021 season) and the Mirador series (Alfisols) (orchard in the 2021–2022 season), with loamy and loamy-clayey textures, respectively [61]. The orchards were managed with a fertilization program, irrigation, insect control, and weed control implemented by the company. The trials were established on 30 September 2020 (first season) and 13 October 2021 (second season). The experiment used a factorial arrangement in a completely randomized block design with four replications. Treatments were applied to five trees per plot, arranged in two rows separated by an untreated row to prevent drift. Evaluation was focused on the central tree in each treated row to ensure no contamination from adjacent treatments due to potential wind drift.

#### 4.5.2. Fungal Inoculations in Hazelnut Trees

Five healthy branches per tree were selected, and a hole approximately 4 mm deep and 6.5 mm in diameter was made using an electric drill (Bauker^®^, SD-GS1041, Suzhou, China) [62,63]. The five trees per plot of each repetition were sprayed individually with the chemical and biological fungicide treatments described in Table 6, which were selected from the previous experiments, using an experimental adjustable static piston sprayer. An average volume of 2.5 L per plant for each application (1000 L per hectare) was used, and the trees were sprayed until leaf runoff. One hour after the treatments were applied, the fungus was inoculated into the holes of the branches using 5 mm diameter discs of actively growing *D. mutila* mycelium, ensuring contact with the internal tissues of the wood (vascular cambium). For each treatment, a branch was inoculated with a sterile PDA disc as a control (Figure S2). Finally, the holes were sealed with plastic film to protect the lesion from external contaminants and improve moisture conditions for phytopathogen growth. The inoculation methodology was repeated the following day on two additional branches per tree that had already been drilled and sprayed with the treatments the previous day.

Additionally, seven days after fungicide application, the diameter of the inoculation zone of each branch was measured with a Peltier caliper (Ubermann, 6” stainless, China) to determine its influence as a covariate on the length of the vascular lesion.

The inoculated branches were removed from the orchards after seven months in the first season and after five months in the second season for evaluation. A vertical cut was made through the vascular tissue that passed through the inoculation point on the branch to measure the length of the necrotic lesion from end to end. Subsequently, the edge of the necrotic area observed in the inoculated branches was cut, and the surface was disinfected for 1 min in a 2.5% sodium hypochlorite solution, followed by two washes for 1 min with SDW. Isolations were carried out by placing a piece of necrotic tissue on PDA. The plates were incubated in the dark at 25 °C for 10 days. *D. mutila* was identified through morphological observations under an optical microscope. For each plant, an inoculated branch was selected to assess the number of male (catkins) and female (glomerulus) flowers, which were compared with uninoculated branches within each experimental plot. The evaluation was conducted at BBCH 55 (male flowers) and BBCH 600 (female flowers) in the first season, while in the second season, male flowers were evaluated at BBCH 57 and female flowers at BBCH 600 [64].

Throughout the experiment, the environmental conditions of the area were monitored monthly, using data from the meteorological station of Frutícola Agrichile S.A., San Gregorio, Ñiquén, Ñuble Region.

### 4.6. Data Analysis

The results of the evaluations conducted in the in vitro, pot, and field experiments were subjected to normality and homogeneity of variance analysis using the Shapiro–Wilk and Levene tests, respectively. If assumptions were fulfilled, the data were analyzed by analysis of variance (ANOVA); otherwise, the non-parametric Kruskal–Wallis test was used. Means were compared using the Fisher’s Least Significant Difference (LSD) test (α = 0.05). All the statistical analyses were conducted using Infostat software (Universidad de Córdoba, Argentina) version 2022 [65].

## 5. Conclusions

*Bacillus subtilis* QST 713, *Pseudomonas protegens* ChC7, the consortium of *Bionectria ochroleuca* Mitique, *Trichoderma gamsii* Volqui, and *Hypocrea virens* Ñire were selected as effective BCAs to inhibit the development of *Diplodia mutila* mycelium in the in vitro assays. The application of the selected chemical fungicides and BCAs on the wounds subsequently inoculated with *D. mutila* mycelium in hazelnut wood resulted in significant reductions in wood necrosis in nursery plants, whereas field trials demonstrated that systemic fungicides fluopyram/tebuconazole, fluxapyroxad/pyraclostrobin, and tebuconazole and the three selected products based on BCAs were effective in reducing necrosis in inoculated wounds in branches, but BCA efficacy against *D. mutila* varied between seasons. The chemical and biological fungicides assessed in this research are options of control strategies to reduce the incidence of *D. mutila* in hazelnut production, and thus they may be included in future integrated disease management programs.

## Figures and Tables

**Table 1:** Degree of inhibition of in vitro mycelial growth of each fungicide on *Diplodia mutila* determined by mycelium development categories in the Petri dish at different incubation times.

	Incubation Time		
Fungicide Group/Active Ingredient	48 h	5 Days	14 Days
(B1) Methyl benzimidazole carbamate (MBC) fungicides			
Methyl thiophanate	(++++) ^1^	(++++)	(++)
(C3) Quinone outside inhibitor (QoI) Fungicides			
Pyraclostrobin	(++)	(−)	(−)
Azoxystrobin/Difenoconazole	(++)	(+)	(−)
Trifloxystrobin	(+)	(−)	(−)
Kresoxim methyl/Miclobutanil	(++)	(−)	(−)
Kresoxim methyl/Tebuconazole	(+++)	(+)	(−)
(C2) Succinate dehydrogenase inhibitor (SDHI) fungicides			
Penthiopyrad	(++)	(−)	(−)
Fluxapyroxad/Pyraclostrobin	(+++)	(++)	(++)
Fluopyram/Tebuconazole	(+++)	(++)	(++)
(C) Not grouped			
Fluazinam	(++++)	(+++)	(++)
(D1) Anilinopyrimidine (AP) fungicides			
Pyrimethanyl/Difenoconazole	(++)	(−)	(−)
(G1) Demethylation inhibitor (DMI) fungicides (SBI: Class I)			
Difenoconazole ^2^	(++)	(+)	(−)
Difenoconazole ^3^	(++)	(−)	(−)
Difenoconazole ^4^	(++)	(+)	(−)
Miclobutanil	(++)	(−)	(−)
Difenoconazole/Kresoxim methyl	(++)	(−)	(−)
Tebuconazole	(+++)	(++)	(++)
Prochloraz	(+++)	(+++)	(++)
(M) Dithiocarbamates and relatives (electrophiles)			
Mancozeb	(++++)	(−)	(−)
Natural extracts & salts			
Potassium Hydrogenicarbonate	(−)	(−)	(−)
Extracts of *Quillaja saponaria* and *Yucca schidigera*	(−)	(−)	(−)
Plant extracts and natural fatty acids	(+++)	(−)	(−)

^1^ Mycelium development on PDA medium: (−) = normal fungal growth, (+) = moderate fungal growth (1% to 30% less than the normal fungal growth), (++) = mild fungal growth (31% to 75% less fungal growth), (+++) = limited fungal growth (76% to 99%), and (++++) = 100% inhibition of fungal growth. ^2^ Difenoconazol 25 EC, Agrospec^®,^ Maipú, Chile; ^3^ Dominio 25 EC, Anasac, Lampa, Chile; ^4^ Score^®^ 250 EC, Syngenta, Monthey, Swiss.

**Table 2:** Mycelial inhibition percentage from different bacterial strains with antagonistic activity against fungus *Diplodia mutila* after seven days of incubation under in vitro conditions. Different letters indicate significant differences between treatments according to the non-parametric Kruskal–Wallis test (*p* ≤ 0.05).

Bacterial Strain	Percentage of Mycelial Inhibition
*Pseudomonas protegens* Ca2	41.4 abc
*Pseudomonas protegens* Ca6	37.9 ab
*Pseudomonas protegens* ChC7	45.3 bc
*P. protegens* strains Ca2 + Ca6 + ChC7	42.2 abc
*Pantoea* sp. AP113	6.3 a
*Bacillus subtilis* QST 713	51.9 c
Interquartile range (IQR)	28.2
*p*-value	0.02

**Table 3:** Mean length values of necrotic lesions caused by *Diplodia mutila* on branches of hazelnut plants cv. Barcelona and Tonda di Giffoni (average of both cultivars), sprayed with different fungicides and inoculated immediately after application under controlled pot conditions. Different letters indicate statistical differences between treatments according to Fisher’s LSD test (α = 0.05).

Treatments	Mean Length (mm) of Necrotic Lesions in Inoculated Branches
Water control	50.9 d
*Bacillus subtilis* QST 713	33.9 bc
*Bionectria ochroleuca* Mitique *Trichoderma gamsii* Volqui *Hypocrea virens* Ñire	23.1 ab
*Pseudomonas protegens* ChC7	26.9 bc
Fluazinam	23.9 ab
Fluopyram/Tebuconazole	25.7 abc
Fluxapyroxad/Pyraclostrobin	19.9 a
Prochloraz	34.6 c
Tebuconazole	31.1 bc
Coefficient of variation	9.5
*p*-value	<0.01

**Table 4:** Mean length values of necrotic lesions caused by the fungus *Diplodia mutila* on branches of hazelnut cv. Tonda di Giffoni, with inoculations on the same day and 24 h after fungicide application, under field conditions in the 2020–2021 season. Row with different letters indicate statistical differences between inoculation times according to Fisher’s LSD test (α = 0.05).

	Mean Length of Necrotic Lesions (mm)	
Treatments	Inoculation on the Day of Fungicide Application	Inoculation 24 h after Fungicide Application
Control	48.1	45.4
Fluazinam	46.7	45.6
Fluopyram/Tebuconazole	60.7	36.0
Fluxapyroxad/Pyraclostrobin	45.0	32.5
Prochloraz	45.4	41.5
Tebuconazole	50.9	39.3
Average	49.5 B	40.1 A
Coefficient of variation	6.34	
*p*-value Inoculation time	0.008	

**Table 5:** Mean length values of necrotic lesions caused by the fungus *Diplodia mutila* on branches of hazelnut cv. Tonda di Giffoni with inoculations on the same day of applications and 24 h after fungicide application, under field conditions in the 2021–2022 season. Different lowercase letters indicate significant differences between treatments, and different uppercase letters indicate significant differences between inoculation times within each treatment, according to Fisher’s LSD test (α = 0.05).

	Mean Length of Necrosis (mm)			
Treatments	Inoculation on the Day of Fungicide Application		Inoculation 24 h after Fungicide Application	
Control	71.8 c	A	123.8 b	B
Fluazinam	60.2 bc	A	108.6 b	B
Fluopyram/Tebuconazole	45.3 a	A	87.8 a	B
Fluxapyroxad/Pyraclostrobin	48.7 a	A	80.8 a	B
Penthiopyrad	45.5 a	A	121.8 b	B
Prochloraz	54.3 ab	A	113.0 b	B
Tebuconazole	47.9 a	A	76.9 a	B
Average	72.2		101.8	
Coefficient of variation	2.75			
*p*-value T × I	<0.01			

**Table 6:** Chemical fungicides and biological control agents selected in the in vitro assays and further evaluated under controlled and field conditions.

Active Ingredient	Formulation	Trade Name	Manufacturer	Commercial Dose
Fluazinam	500 g L^−1^ SC	Shirlan^®^ 500	Syngenta	75 mL hL^−1^ (Apple trees)
Fluopyram/Tebuconazole	200/200 g L^−1^ SC	Luna^®^ Experience 400	Bayer	40 mL hL^−1^ (Stone and pome trees)
Fluxapiroxad/Pyraclostrobin	250/250 g L^−1^ SC	Elmus^®^	BASF	40 mL hL^−1^ (Stone and pome trees)
Penthiopyrad	200 g L^−1^ SC	Fontelis^®^	Corteva	40 mL hL^−1^ (Apple trees)
Prochloraz	400 g L^−1^ EC	Mirage 40%	Adama	120 mL hL^−1^ (Wheat and barley)
Tebuconazole	430 g L^−1^ SC	Tebuconazole 430	Agrospec	40 mL hL^−1^ (Stone trees)
*Bacillus subtilis* QST 713	13,68 g L^−1^ SC (1 × 10^9^ cfu g^−1^)	Serenade^®^ ASO	Bayer	600 mL hL^−1^ (Stone and pome trees)
*Bionectria ochroleuca* Mitique *Trichoderma gamsii* Volqui *Hypocrea virens* Ñire	10/10/10 g kg^−1^ WG (3 × 10^8^ cfu g^−1^)	Mamull^®^	Bio Insumos Nativa	100 g hL^−1^ (Stone and pome trees)
*Pseudomonas protegens* ChC7	1 × 10^7^	N/D	N/D	1000 mL hL^−1^

N/D = Name not defined.

## Data Availability

The data presented in this study are available on request from the corresponding author.

## Supplementary Materials

The following supporting information can be downloaded at https://www.mdpi.com/article/10.3390/plants13192753/s1: Table S1: Chemical fungicides, plant extracts and salts, and biological control agents used to select control strategies against fungus *Diplodia mutila* under in vitro conditions. Table S2: Density of measured catkins and glomerulus evaluated in branches inoculated and non-inoculated with *Diplodia mutila* under field conditions in the 2020–2021 and 2021–2022 seasons. Table S3: Active ingredients tested for inhibitory activity against *Diplodia mutila*, trade names, and target diseases and crops according to the manufacturer’s recommendation. Table S4: Trade name, active ingredients (ppm), concentration suggested by the manufacturer, and commercial dose of the different fungicides applied in hazelnut plants (expressed in mg in 300 µL) to evaluate inhibitory activity against *Diplodia mutila* under controlled and field experiments. Figure S1: Grade scale^1^ with five criteria for the development of *Diplodia mutila* mycelium on plates with PDA medium treated with the commercial dose of each chemical fungicide used in the preselection trial. Figure S2: Methodology of the experiment in a commercial orchard of hazelnut cv. Tonda di Giffoni: branch perforation using a drill, application of treatments on the entire plant, and inoculation of a *Diplodia mutila* disc into the vascular tissue of the drilled hazelnut branch. Figure S3: Inoculation experiment of *Diplodia mutila* with localized treatment applications on the stems of potted hazelnut plants (cv. Tonda di Giffoni and Barcelona), conducted under a Raschel net. Figure S4: Necrotic lesions observed on the stems of potted hazelnut plants inoculated with *Diplodia mutila* after localized application of different treatments in the inoculation site. (A) Control (water); (B) *Bacillus subtilis* strain QST 713 [Serenade ASO, Bayer]; (C) *Bionectria ochroleuca* Mitique, *Trichoderma gamsii* Volqui, *Hypocrea virens* Ñire [Mamull, Bio Insumos Nativa SpA]; (D) *Pseudomonas protegens* ChC7; (E) Fluazinam; (F) Fluopyram/Tebuconazole; (G) Fluxapyroxad/Pyraclostrobin; (H) Prochloraz; (I) Tebuconazole. Figure S5: Necrotic lesions observed on branches of hazelnut cv. Tonda di Giffoni inoculated with *Diplodia mutila* in the control treatment (water), including samples of inoculations performed on the same day and 24 h· after fungicide application, under field conditions during the first season. Figure S6: Necrotic lesions observed on branches of hazelnut cv. Tonda di Giffoni inoculated with *Diplodia mutila* on the same day as fungicide application, under field conditions during the second season. (A) Control; (B) Fluazinam; (C) Fluopyram/Tebuconazole; (D) Fluxapyroxad/Pyraclostrobin; (E) Penthiopyrad; (F) Prochloraz; (G) Tebuconazole. Figure S7: Necrotic lesions observed on branches of hazelnut cv. Tonda di Giffoni inoculated with *Diplodia mutila* 24 h after fungicide application, under field conditions during the second season. (A) Control; (B) Fluazinam; (C) Fluopyram/Tebuconazole; (D) Fluxapyroxad/Pyraclostrobin; (E) Penthiopyrad; (F) Prochloraz; (G) Tebuconazole. Figure S8: Necrotic lesions observed on branches of hazelnut cv. Tonda di Giffoni inoculated with *Diplodia mutila* after the application of antagonists, under field conditions during the first season. (A) Control (water); (B) *Bacillus subtilis* strain QST 713 [Serenade ASO, Bayer]; (C) *Bionectria ochroleuca* Mitique, *Trichoderma gamsii* Volqui, *Hypocrea virens* Ñire [Mamull, Bio Insumos Nativa SpA]; (D) *Pseudomonas protegens* ChC7. Figure S9: Necrotic lesions observed on branches of hazelnut cv. Tonda di Giffoni inoculated with Diplodia mutila after the application of antagonists, under field conditions during the second season. (A) Control (water); (B) Bacillus subtilis strain QST 713 [Serenade ASO, Bayer]; (C) Bionectria ochroleuca Mitique, Trichoderma gamsii Volqui, Hypocrea virens Ñire [Mamull, Bio Insumos Nativa SpA]; (D) Pseudomonas protegens ChC7. Figure S10: Daily records of precipitation and average temperatures obtained from the Red Agroclimática Nacional database (AGROMET) in the 2020–2021 (A) and 2021–2022 (B) seasons, measured in the Ñiquén meteorological station, Ñuble region. Horizontal lines indicate favourable temperatures for Diplodia mutila mycelial growth (Chen et al., 2020).
