# Supplementary material for: Assessment of Chemical and Biological Fungicides for the Control of Diplodia mutila Causing Wood Necrosis in Hazelnut
Source: Plants (Basel). 2024 Sep 30;13(19):2753. doi: 10.3390/plants13192753 (PMC11478353; doi:10.3390/plants13192753)
Supplement: Supplementary file 1 [file plants-13-02753-s001.zip › 01.Supplementary Material Additional tables and grade scale.pdf]

Supplementary Material: Additional tables and grade scale

Table S1: Chemical fungicides, plant extracts and salts, and biological control agents used to select control strategies against fungus *Diplodia mutila* under *in vitro* conditions.

Chemical group	Active ingredient (a.i.)	Mode of action	Trade name	Manufacturer	Dose
**(B) MBC (methyl benzimidazole carbamate) fungicides**					
Thiophanates	Methyl thiophanate	Systemic	Cercobin® M	BASF	85 g hL^{-1}
**(C) Quinone outside inhibator (QoI) fungicide**					
Methoxycobamate	Pyraclostrobin	Translaminar	Comet® WG	BASF	50 g hL^{-1}
Methoxyyacrylate Triazole	Azoxystrobin/ Difenoconazole	Contact/ Systemic	Amistar® Top SC	Syngenta	40 mL hL^{-1}
Oxymino Acetate	Trifloxystrobin	Translaminar	Flint 50% WG	Bayer	10 g hL^{-1}
Oxymino Acetate/Triazole	Kresoxim methyl/ Miclobutanil	Contact/ Systemic	Silver SC	Anasac	70 mL hL^{-1}
Oxymino Acetate/Triazole	Kresoxim methyl/ Tebuconazole	Contact/ Systemic	Fungifull® 275 SC	Agrospec	65 mL hL^{-1}
**(C) SDHI (succinate dehydrogenase inhibitor) fungicides**					
Pyrazole-4-carboxamide	Penthiopyrad	Systemic	Fontelis®	Dupont	40 mL hL^{-1}
Pyrazole-4-carboxamide/Methoxyarbamate	Fluxapiroxad/ Pyraclostrobin	Systemic/ Translaminar	Elmus ®	BASF	45 mL hL^{-1}
Pyridinyl-ethyl-benzamide/Triazole	Fluopyram/ Tebuconazole	Translaminar/ Systemic	Luna® Experience 400 SC	Bayer	45 mL hL^{-1}
**(C) Not grouped**					
Dinitroaniline	Fluazinam	Contact	Shirlan® 500 SC	Syngenta	75 mL hL^{-1}
**(D) AP (anilinopyrimidine) fungicides**					
Anilinopyrimidine/ Triazole	Pyrimethanyl/ Difenoconazole	Contact/Systemic	Marcial SC	Anasac	60 mL hL^{-1}
**(G) DMI (demethylation inhibitor) fungicides (SBI: Class I)**					
Triazole	Difenoconazole	Systemic	Difenoconazole 25 EC	Agrospec	15 mL hL^{-1}
Triazole	Difenoconazole	Systemic	Dominio 25 EC	Anasac	15 mL hL^{-1}
Triazole	Difenoconazole	Systemic	Score® 250 EC	Syngenta	15 mL hL^{-1}
Triazole	Miclobutanil	Systemic	Miclobutanil 40 WP	Agrospec	10 g hL^{-1}
Triazole/Oximino Acetate	Difenoconazole/Kresoxim methyl	Systemic/ Contact	Hit 140 EC	Agrospec	75 mL hL^{-1}
Triazole	Tebuconazole	Systemic	Tebuconazole 430 SC	Agrospec	30 mL hL^{-1}
Imidazole	Prochloraz	Contact	Mirage 40% EC	Adama	120 mL hL^{-1}
**(M) Dithiocarbamates and relatives (electrophiles)**					
Dithiocarbamate	Mancozeb	Contact	Manzate	Anasac	210 g hL^{-1}
**(BM) Microbial (live microbial strains or extract, metabolites)**					

Antagonist bacteria	*Pseudomonas protegens*	Ca2+Ca6+ChC7	UdeC[1]	1000 mL hL^{-1}
Antagonist bacteria	*Pseudomonas protegens* Ca2	Ca2	UdeC	1000 mL hL^{-1}
Antagonist bacteria	*Pseudomonas protegens* Ca6	Na6	UdeC	1000 mL hL^{-1}
Antagonist bacteria	*Pseudomonas protegens* ChC7	ChC7	UdeC	1000 mL hL^{-1}
Antagonist bacteria	*Pantoea* spp.	Pantoea	UdeC	1000 mL hL^{-1}
Antagonist bacteria	*Bacillus subtilis* QST 713	Serenade ASO	Bayer	800 mL hL^{-1}
Antagonistic fungus and bacteria	*Trichoderma spp. and* Bacillus *spp.*	Puelche -VTO	Bio Insumos Nativa	125 g hL^{-1}
Antagonist fungus	*Bionectria ochroleuca* Mitique, *Trichoderma gamsii* Volqui, *Hypocrea virens* Ñire	Mamull WG	Bio Insumos Nativa	100 g hL^{-1}
**Natural extracts & salts**				
Inorganic salts	Potassium Hydrogenicarbonate	Kaligreen®	SummitAgro	200 g hL^{-1}
Saponins	Extracts of *Quillaja saponaria* and *Yucca schidigera*	GrowActive	Desert king	200 mL hL^{-1}
Saponins	Plant extracts and natural fatty acids	Pruning paste	Phytological	Undiluted

[1] Bacteria available at the Laboratory of Phytopathology of the Universidad de Concepción (UdeC), Chillán campus, Chile.

Figure S1: Grade scale[1] with five criteria for the development of *Diplodia mutila* mycelium on plates with PDA medium treated with the commercial dose of each chemical fungicide used in the preselection trial.

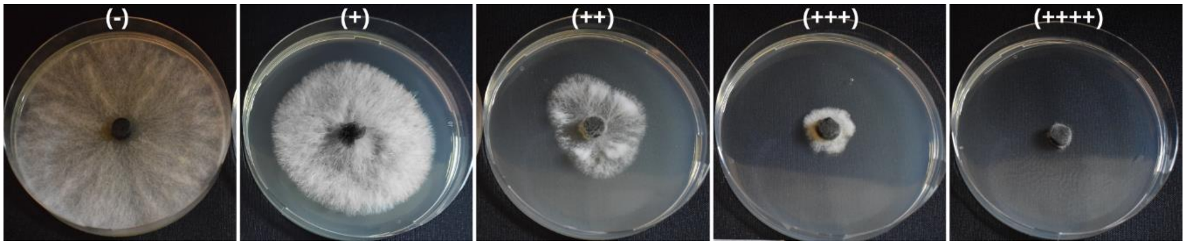

[1] (-) = Normal fungal growth, (+) = Moderate fungal growth (1 to 30% less than the normal fungal growth), (++) = Mild fungal growth (31 to 75% less fungal growth), (+++) = Limited fungal growth (76 to 99%), and (++++) = 100% inhibition of fungal growth.

Table S2: Density of measured catkins and glomerulus evaluated in branches inoculated and non-inoculated with *Diplodia mutila* under field conditions in the 2020-21 and 2021-22 seasons.

	Floral density (Number of flowers cm^{-2})			
	2020-21 season		2021-22 season	
Treatments	Catkins	Glomerulus	Catkins	Glomerulus
Non-inoculated control	0.62	0.21	0.81	0.79
Inoculated control	0.54	0.19	0.71	0.60
Bacillus subtilis QST 713	0.67	0.15	0.54	0.50
Bionectria ochroleuca strain Mitique<br>*Trichoderma gamsii* strain Volqui<br>*Hypocrea virens* strain Ñirre	0.77	0.16	0.58	0.61
Pseudomonas protegens ChC7	0.61	0.16	0.57	0.58
Fluazinam	0.74	0.14	0.45	0.67
Fluopyram/Tebuconazole	0.52	0.16	0.64	0.71
Fluxapyroxad/Pyraclostrobin	0.49	0.08	0.76	0.65
Penthiopyrad	n/a[1]	n/a	0.63	0.67
Prochloraz	0.41	0.16	0.70	0.67
Tebuconazole	0.61	0.19	0.52	0.53
Interquartile range	0.34	0.10	0.40	0.22
P-value	0.93	0.28	0.69	0.43

[1] n/a = not assessed.

TableS3. Active ingredients tested for inhibitory activity against *Diplodia mutila*, trade names, and target diseases and crops according to the manufacturer's recommendation.

Active ingredient	Trade name	Diseases	Crops
Fluazinam	Shirlan® 500 SC	Gray mold, apple scab, moldy core, late blight, white mold, others fungus.	Grapevine, apple, potato, beet, berries, tulip and lilium.
Fluopyram/ Tebuconazole	Luna® Experience 400 SC	Powdery mildew, gray mold, brown rot, apple scab, moldy core, early blight, leek rust.	Vines, stone fruit, apple, pear, olive, tomato, potato, curcubits.
Fluxapiroxad/ Pyraclostrobin	Elmus ®	Gray mold, brown rot, powdery mildew, apple scab, moldy core, botryosphaeria dieback, onion leaf blight.	Stone fruit, apple, pear, walnut, blueberry, tomato, onion.
Penthiopyrad	Fontelis ®	Alternaria blight, gray mold, apple scab, powdery mildew, brown apical necrosis (BAN), early blight, white mold.	Cherry, rose, apple, walnut, tomato, potato, lettuce.
Prochloraz	Mirage 40% EC	Anthracnose, white mold, gray mold, fusarium head blight, powdery mildew, stem-end rot, Phoma leaf spot.	Lupine, cereals, avocado, canola.
Tebuconazole	Tebuconazole 430 SC	Powdery mildew, gray mold, brown rot, blossom blight, sour rot.	Grapevine, stone fruit, pine, eucalyptus.

TableS4. Trade name, active ingredients (ppm), concentration suggested by the manufacturer, and commercial dose of the different fungicides applied in hazelnut plants (expressed in mg in 300 μL) to evaluate inhibitory activity against *Diplodia mutila* under controlled and field experiments.

Trade name	Active ingredient	Formulation	Manufacturer	Concentration	ppm	mg in 300 μL
Shirlan® 500 SC	Fluazinam	500 g L^{-1}	Syngenta	75 mL hL^{-1}	375 ppm	0.11
Luna® Experience 400 SC	Fluopyram/ Tebuconazole	200 g L^{-1}; 200 g L^{-1}	Bayer	40 mL hL^{-1}	160 ppm	0.05
Elmus ®	Fluxapiroxad/ Pyraclostrobin	250 g L^{-1}; 250 g L^{-1}	BASF	40 mL hL^{-1}	200 ppm	0.06
Fontelis ®	Penthiopyrad	200 g L^{-1}	Corteva	40 mL hL^{-1}	80 ppm	0.02
Mirage 40% EC	Prochloraz	400 g L^{-1}	Adama	120 mL hL^{-1}	480 ppm	0.14
Tebuconazole 430 SC	Tebuconazole	430 g L^{-1}	Agrospec	40 mL hL^{-1}	172 ppm	0.05

Table S5: Daily records of precipitation and average temperatures obtained from the Red Agroclimática Nacional database (AGROMET) in the 2020-2021 (A) and 2021-2022 (B) seasons, measured in the Ñiquén meteorological station, Ñuble region. Horizontal lines indicate favorable temperatures for *Diplodia mutila* mycelial growth (Chen *et al*., 2020).

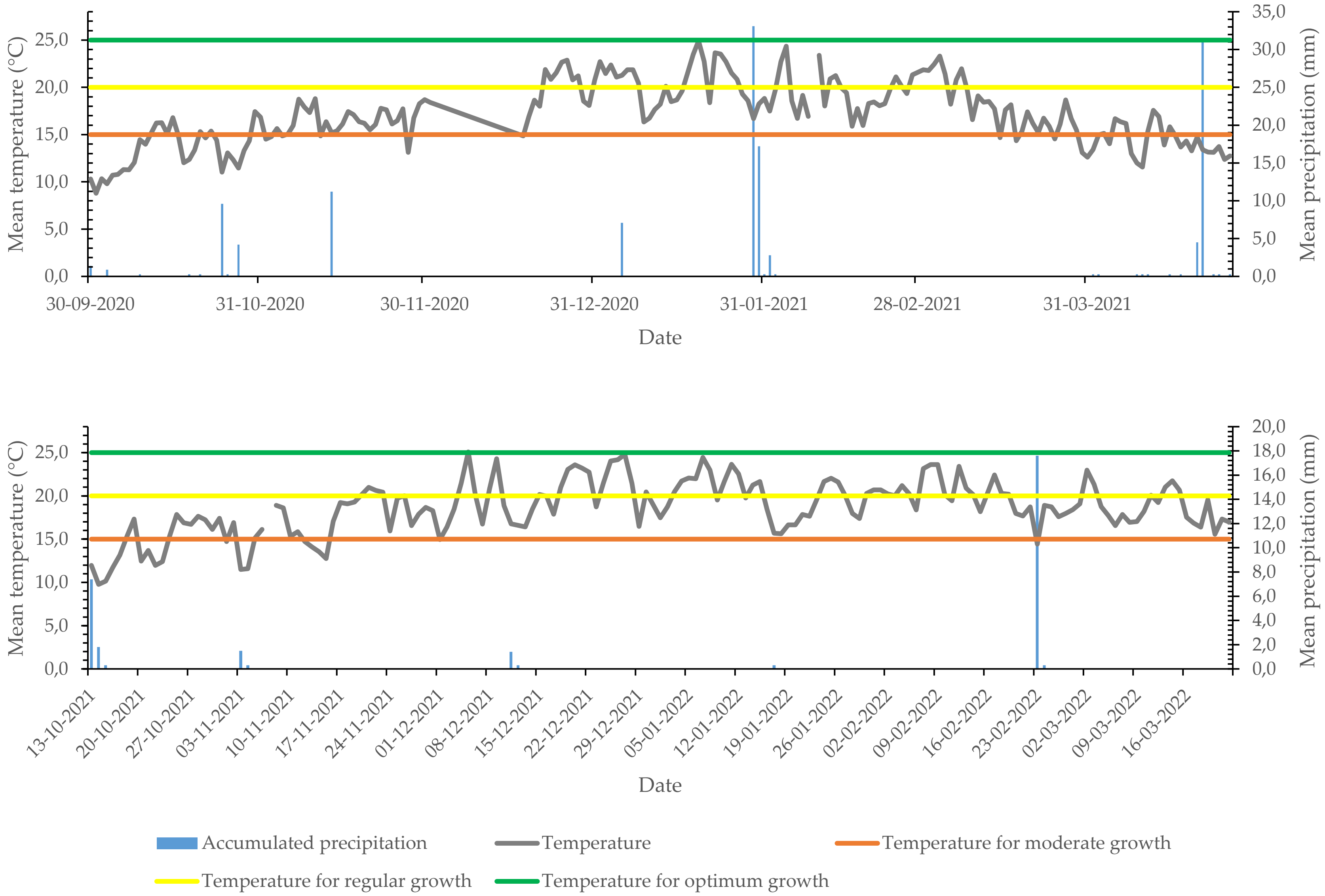

Mean temperature (°C)
Mean precipitation (mm)
Date
30-09-2020
31-10-2020
30-11-2020
31-12-2020
31-01-2021
28-02-2021
31-03-2021
13-10-2021
20-10-2021
27-10-2021
03-11-2021
10-11-2021
17-11-2021
24-11-2021
01-12-2021
08-12-2021
15-12-2021
22-12-2021
29-12-2021
05-01-2022
12-01-2022
19-01-2022
26-01-2022
02-02-2022
09-02-2022
16-02-2022
23-02-2022
02-03-2022
09-03-2022
16-03-2022
Accumulated precipitation
Temperature
Temperature for moderate growth
Temperature for regular growth
Temperature for optimum growth
