# Supplementary material for: Assessment of Chemical and Biological Fungicides for the Control of Diplodia mutila Causing Wood Necrosis in Hazelnut
Source: Plants (Basel). 2024 Sep 30;13(19):2753. doi: 10.3390/plants13192753 (PMC11478353; doi:10.3390/plants13192753)

Supplementary Material: Visual records of the methodology and results in hazelnut plants

Article: Assessment of chemical and biological fungicides for the con-trol of *Diplodia mutila* causing wood necrosis in hazelnut

Authors information: Verónica Retamal [1], Juan San Martín [1], Braulio Ruíz [1], Richard M. Bastías [1], Eugenio Sanfuentes [2], María José Lisperguer [3], Tommaso De Gregorio [4], Matteo Maspero [4] and Ernesto Moya-Elizondo [1,*]

[1] Departamento de Producción Vegetal, Facultad de Agronomía, Universidad de Concepción, Chillán, Chile; veronicaretamal@udec.cl (V.R.); juansanmartinm@udec.cl (J.S.M.); braruiz@udec.cl (B.R.); ribastias@udec.cl (R.M.B.)
[2] Laboratorio de Patología Forestal, Facultad de Ciencias Forestales y Centro de Biotecnología, Universidad de Concepción, Concepción, Chile; esanfuen@udec.cl
[3] Departamento Técnico, Frutícola Agrichile S.A., Curicó, Chile
[4] Agri Competence Centre, Ferrero Hazelnut Company (HCo), Senningerberg, Luxembourg
* Correspondence: emoya@udec.cl

Abstract: Fungal trunk disease (FTD) poses a significant threat to hazelnut (*Corylus avellana* L.) production worldwide. In Chile, the fungus *Diplodia mutila*, from the Botryosphaeriaceae family, has been frequently identified causing this disease in the Maule and Ñuble Regions. However, control measures for *D. mutila* remain limited. This research aimed to evaluate the effectiveness of chemical and biological fungicides against *D. mutila* under *in vitro*, controlled pot experiment, and field conditions. An *in vitro* screening of 30 fungicides was conducted. The effectiveness was assessed by measuring the length of vascular lesions in hazelnut branches inoculated with *D. mutila* mycelium disks under controlled and field conditions. Field trials were conducted in a hazelnut orchard in Ñiquén, Ñuble Region, Chile. Results showed that three biological and five chemical fungicides were selected *in vitro* with > 31% inhibition after 14 days. In pot experiments, all fungicides reduced necrotic lesions on branches by 32 to 61%. In field experiments, the most effective systemic fungicides were fluopyram/tebuconazole, fluxapyroxad/pyraclostrobin, and tebuconazole, while the effectiveness of antagonists *Pseudomonas protegens* ChC7 and *Bacillus subtilis* QST713 varied with seasonal temperatures. Effective conventional and biological fungicides against *D. mutila* could be integrated into disease management programs to protect hazelnut wounds from infections.

Figure S1: Grade scale[1] with five criteria for the development of *Diplodia mutila* mycelium on plates with PDA medium treated with the commercial dose of each chemical fungicide used in the preselection trial.

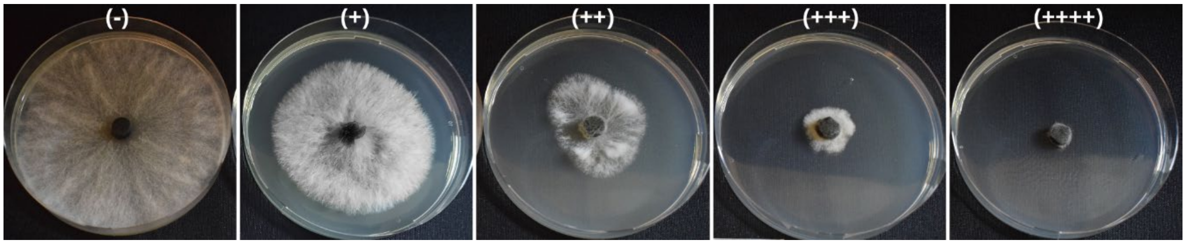

[1] (-) = Normal fungal growth, (+) = Moderate fungal growth (1 to 30% less than the normal fungal growth), (++) = Mild fungal growth (31 to 75% less fungal growth), (+++) = Limited fungal growth (76 to 99%), and (++++) = 100% inhibition of fungal growth.

Figure S2: Methodology of the experiment in a commercial orchard of hazelnut cv. Tonda di Giffoni: branch perforation using a drill, application of treatments on the entire plant, and inoculation of a *Diplodia mutila* disc into the vascular tissue of the drilled hazelnut branch.

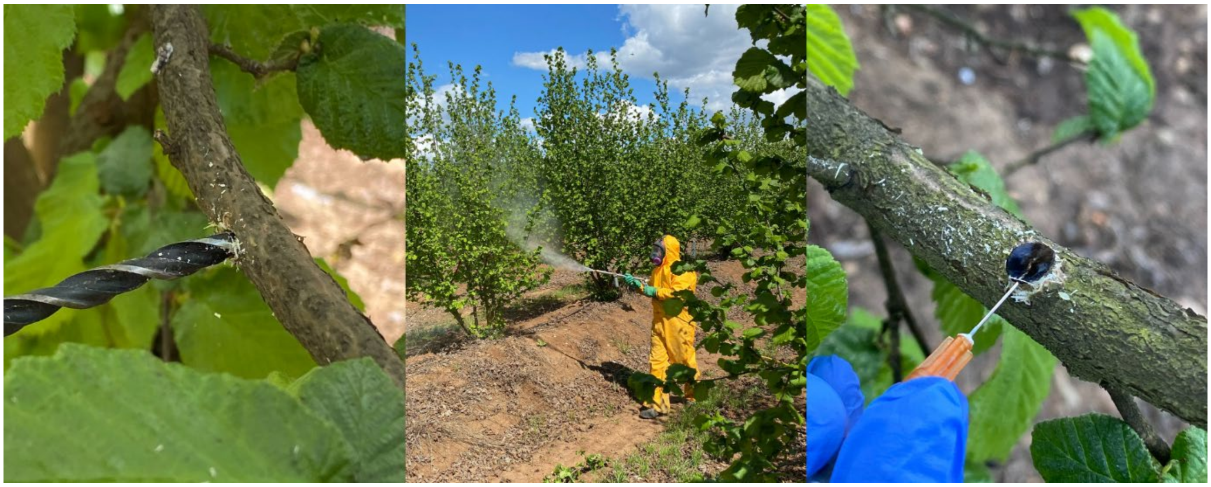

Figure S3: Inoculation experiment of *Diplodia mutila* with localized treatment applications on the stems of potted hazelnut plants (cv. Tonda di Giffoni and Barcelona), conducted under a Raschel net.

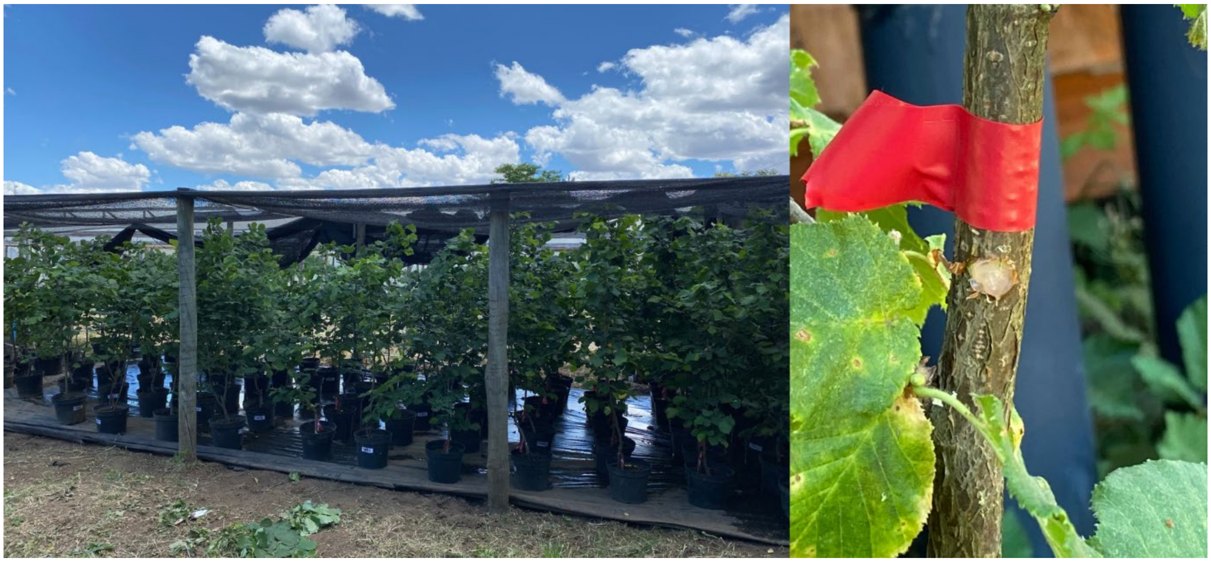

Figure S4: Necrotic lesions observed on the stems of potted hazelnut plants inoculated with *Diplodia mutila* after localized application of different treatments in the inoculation site. (A) Control (water); (B) *Bacillus subtilis* strain QST 713 [Serenade ASO, Bayer]; (C) *Bionectria ochroleuca* Mitique, *Trichoderma gamsii* Volqui, *Hypocrea virens* Ñire [Mamull, Bio Insumos Nativa SpA]; (D) *Pseudomonas protegens* ChC7; (E) Fluazinam; (F) Fluopyram/Tebuconazole; (G) Fluxapyroxad/Pyraclostrobin; (H) Prochloraz; (I) Tebuconazole

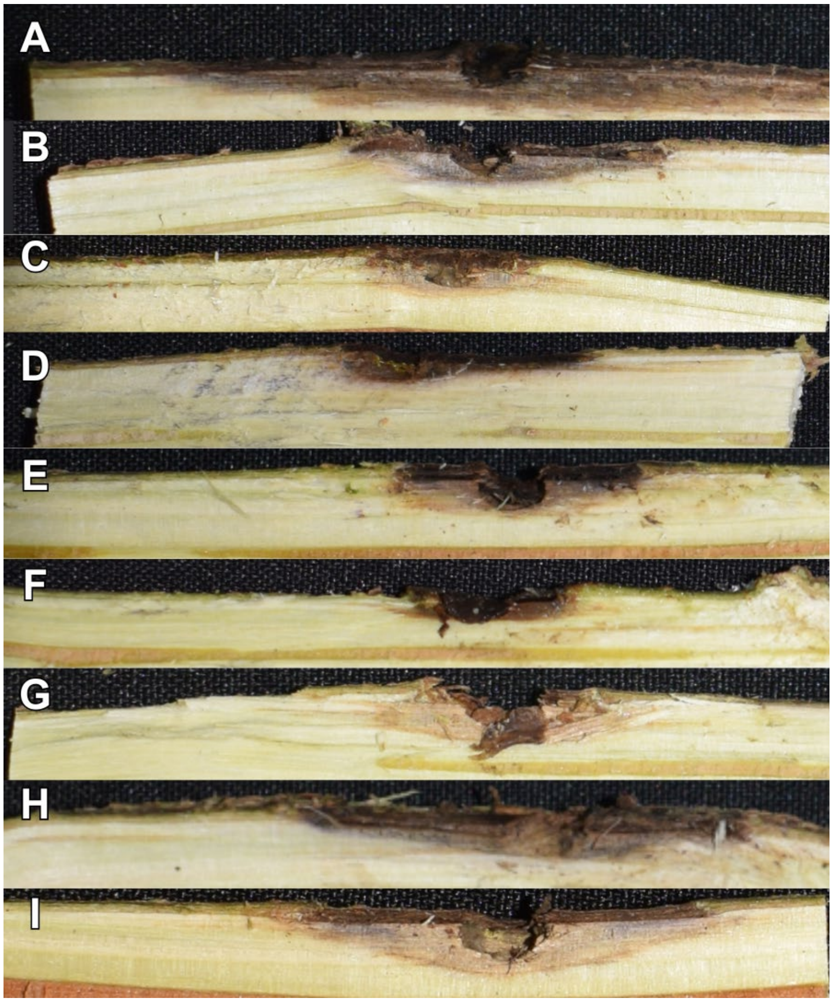

Figure S5: Necrotic lesions observed on branches of hazelnut cv. Tonda di Giffoni inoculated with *Diplodia mutila* in the control treatment (water), including samples of inoculations performed on the same day and 24 hours after fungicide application, under field conditions during the first season.

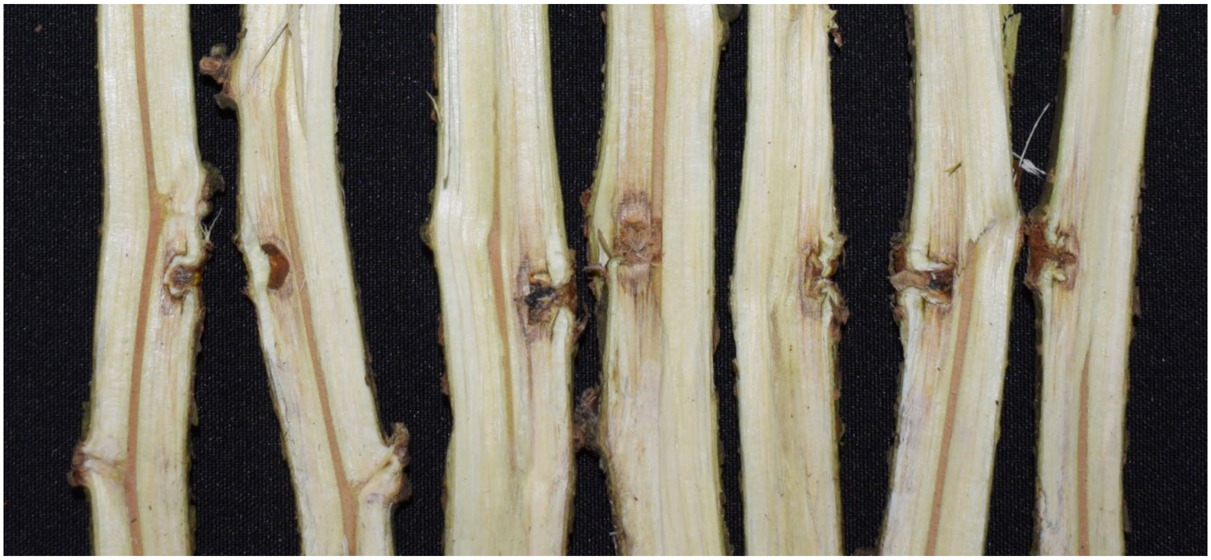

Figure S6: Necrotic lesions observed on branches of hazelnut cv. Tonda di Giffoni inoculated with *Diplodia mutila* on the same day as fungicide application, under field conditions during the second season. (A) Control; (B) Fluazinam; (C) Fluopyram/Tebuconazole; (D) Fluxapyroxad/Pyraclostrobin; (E) Penthiopyrad; (F) Prochloraz; (G) Tebuconazole.

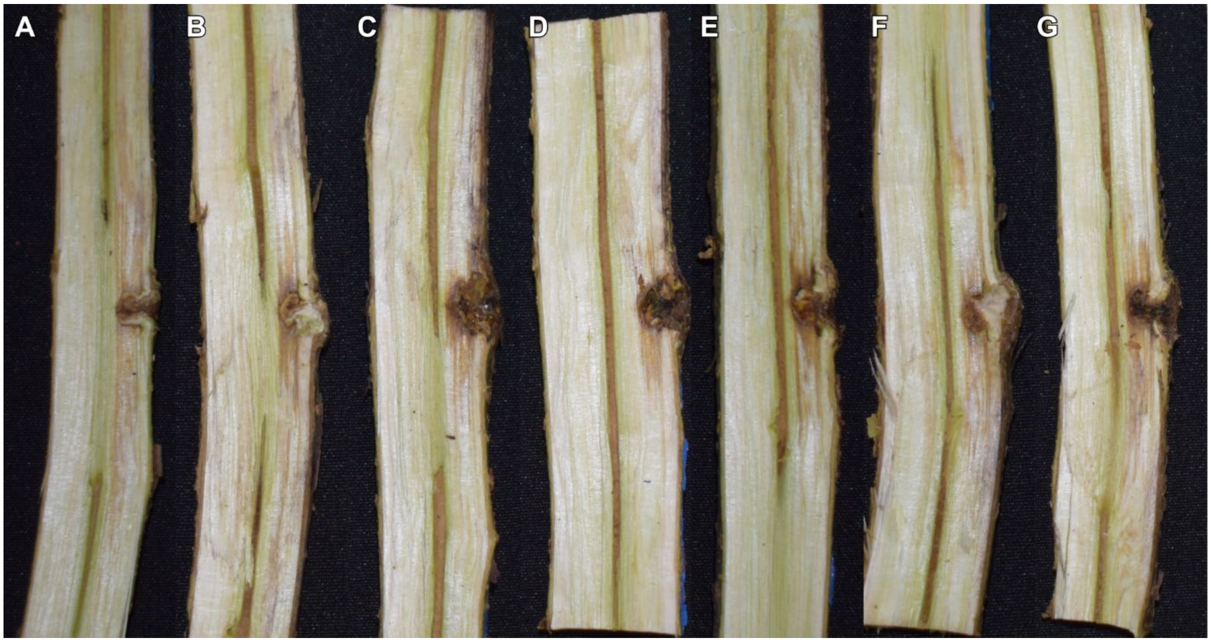

Figure S7: Necrotic lesions observed on branches of hazelnut cv. Tonda di Giffoni inoculated with *Diplodia mutila* 24 hours after fungicide application, under field conditions during the second season. (A) Control; (B) Fluazinam; (C) Fluopyram/Tebuconazole; (D) Fluxapyroxad/Pyraclostrobin; (E) Penthiopyrad; (F) Prochloraz; (G) Tebuconazole.

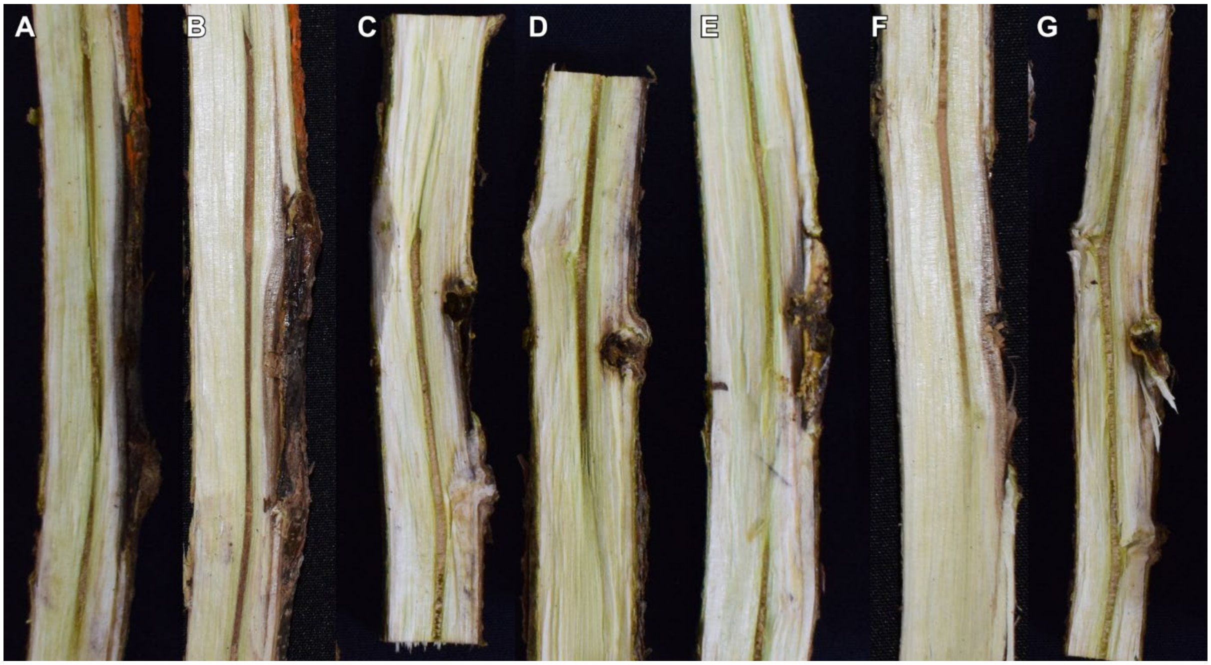

Figure S8: Necrotic lesions observed on branches of hazelnut cv. Tonda di Giffoni inoculated with *Diplodia mutila* after the application of antagonists, under field conditions during the first season. (A) Control (water); (B) *Bacillus subtilis* strain QST 713 [Serenade ASO, Bayer]; (C) *Bionectria ochroleuca* Mitique, *Trichoderma gamsii* Volqui, *Hypocrea virens* Ñire [Mamull, Bio Insumos Nativa SpA]; (D) *Pseudomonas protegens* ChC7.

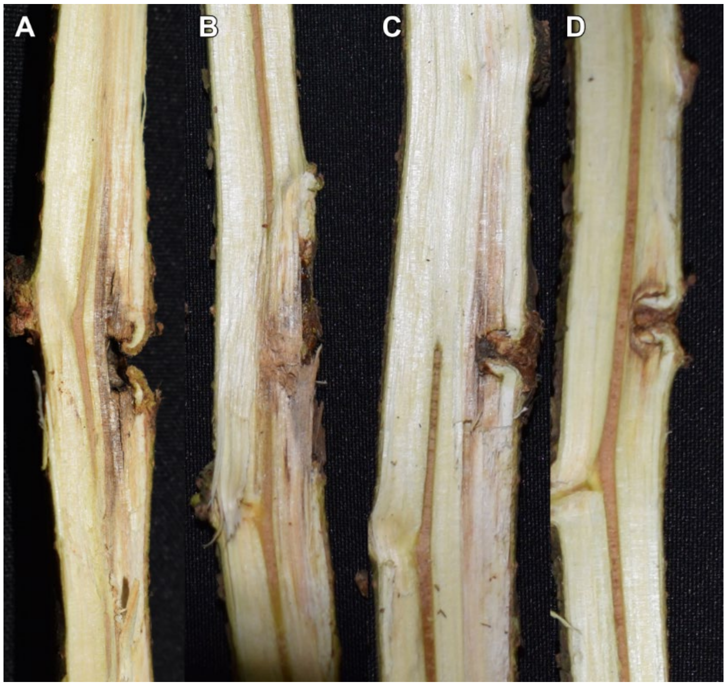

Figure S9: Necrotic lesions observed on branches of hazelnut cv. Tonda di Giffoni inoculated with *Diplodia mutila* after the application of antagonists, under field conditions during the second season. (A) Control (water); (B) *Bacillus subtilis* strain QST 713 [Serenade ASO, Bayer]; (C) *Bionectria ochroleuca* Mitique, *Trichoderma gamsii* Volqui, *Hypocrea virens* Ñire [Mamull, Bio Insumos Nativa SpA]; (D) *Pseudomonas protegens* ChC7.

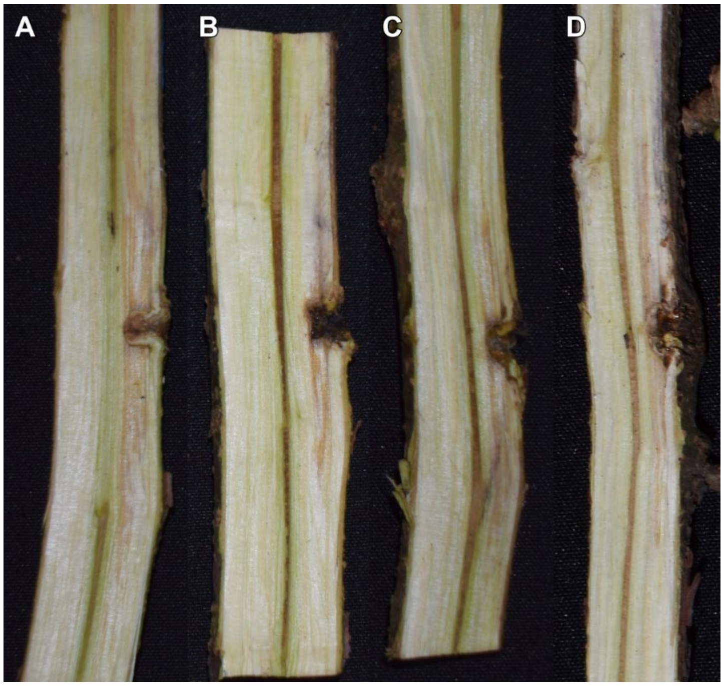

Figure S10: Daily records of precipitation and average temperatures obtained from the Red Agroclimática Nacional database (AGROMET) in the 2020-2021 (A) and 2021-2022 (B) seasons, measured in the Ñiquén meteorological station, Ñuble region. Horizontal lines indicate favourable temperatures for *Diplodia mutila* mycelial growth (Chen *et al*., 2020).

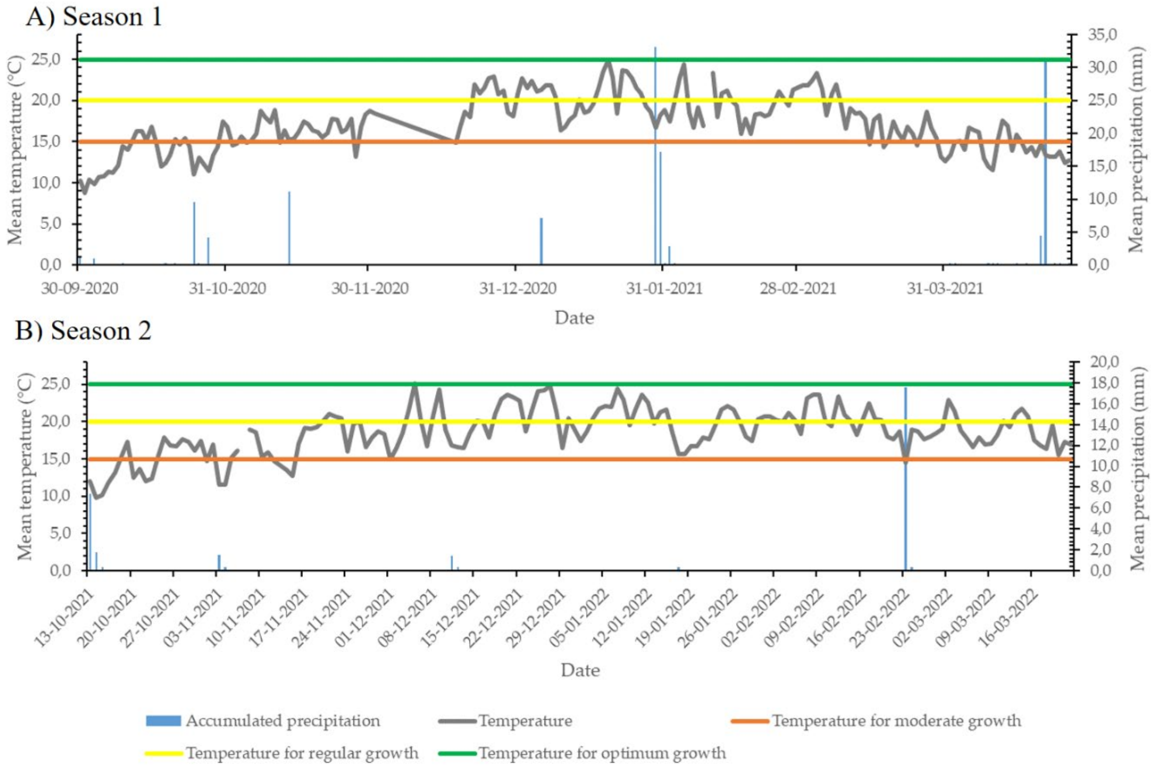

Supplement: Supplementary file 1 [file plants-13-02753-s001.zip › 02.Supplementary Material Visual records of the methodology and results in hazelnut plants mv.pdf]
